# Smokers with CT Detected Emphysema and No Airway Obstruction Have Decreased Plasma Levels of EGF, IL-15, IL-8 and IL-1ra

**DOI:** 10.1371/journal.pone.0060260

**Published:** 2013-04-05

**Authors:** Juan P. de-Torres, David Blanco, Ana B. Alcaide, Luis M. Seijo, Gorka Bastarrika, María José Pajares, Arrate Muñoz-Barrutia, Carlos Ortiz-de-Solorzano, Ruben Pio, Arantza Campo, Usua Montes, Victor Segura, Jesús Pueyo, Luis M. Montuenga, Javier J. Zulueta

**Affiliations:** 1 Pulmonary Department, Clínica Universidad de Navarra, University of Navarra, Pamplona, Spain; 2 Radiology Department, Clínica Universidad de Navarra, University of Navarra, Pamplona, Spain; 3 Centre for Applied Medical Research, University of Navarra, Pamplona, Spain; Universidad Pablo de Olavide, Centro Andaluz de Biología del Desarrollo-CSIC, Spain

## Abstract

**Rationale:**

Low-grade inflammation and emphysema have been shown to be associated with an increased risk of lung cancer. However, the systemic inflammatory response in patients with emphysema is still unknown.

**Objective:**

To compare the plasma cytokine profiles in two groups of current or former smokers without airway obstruction: a control group of individuals without computed tomography (CT) detected emphysema vs. a study group of individuals with CT detected emphysema.

**Methods:**

Subjects underwent a chest CT, spirometry, and determination of EGF, IL-15, IL-1ra, IL-8, MCP-1, MIP-1β, TGFα, TNFα, and VEGF levels in plasma. Cytokine levels in each group were compared adjusting for confounding factors.

**Results:**

160 current smokers and former smokers without airway obstruction participated in the study: 80 without emphysema and 80 subjects with emphysema. Adjusted group comparisons revealed significant reductions in EGF (−0.317, p = 0.01), IL-15 (−0.21, p = 0.01), IL-8 (−0.180, p = 0.02) and IL-1ra (−0.220, p = 0.03) in subjects with emphysema and normal spirometry.

**Conclusions:**

Current or former smokers expressing a well-defined disease characteristic such as emphysema, has a specific plasma cytokine profile. This includes a decrease of cytokines mainly implicated in activation of apoptosis or decrease of immunosurveillance. This information should be taken into account when evaluated patients with tobacco respiratory diseases.

## Introduction

Tobacco smoking is the leading preventable cause of death worldwide [1]. Chronic Obstructive Pulmonary Disease (COPD), lung cancer, and cardiovascular disease will be responsible for the majority of deaths in the developed world in the coming decades [2].

COPD is characterised by chronic airflow limitation caused by a combination of small airway inflammation and parenchymal destruction (emphysema) [3]. Since computed tomography (CT) has been validated as a non-invasive technique capable of identifying areas of lung parenchyma with emphysema [4], several studies have identified a subgroup of apparently healthy smokers who have emphysema but no airway obstruction [5], [6]. Furthermore, this subgroup has the highest risk of developing lung cancer among smokers [5], [6]. It has been proposed that systemic inflammation may play an important pathogenic role in carcinogenesis [7], and whether individuals with emphysema have a different inflammatory profile is unknown. We therefore conducted this cross sectional study to investigate whether, in comparison to smokers without emphysema; patients with emphysema express a different plasma cytokine profile that could explain their increased risk for lung cancer. From a cohort of current and former smokers without airway obstruction participating in a lung cancer screening study, two distinct groups were selected for the purpose of comparing cytokine profiles: a study group of individuals with emphysema on the CT, and a control group of smokers without emphysema.

## Methods

**Figure 1:**
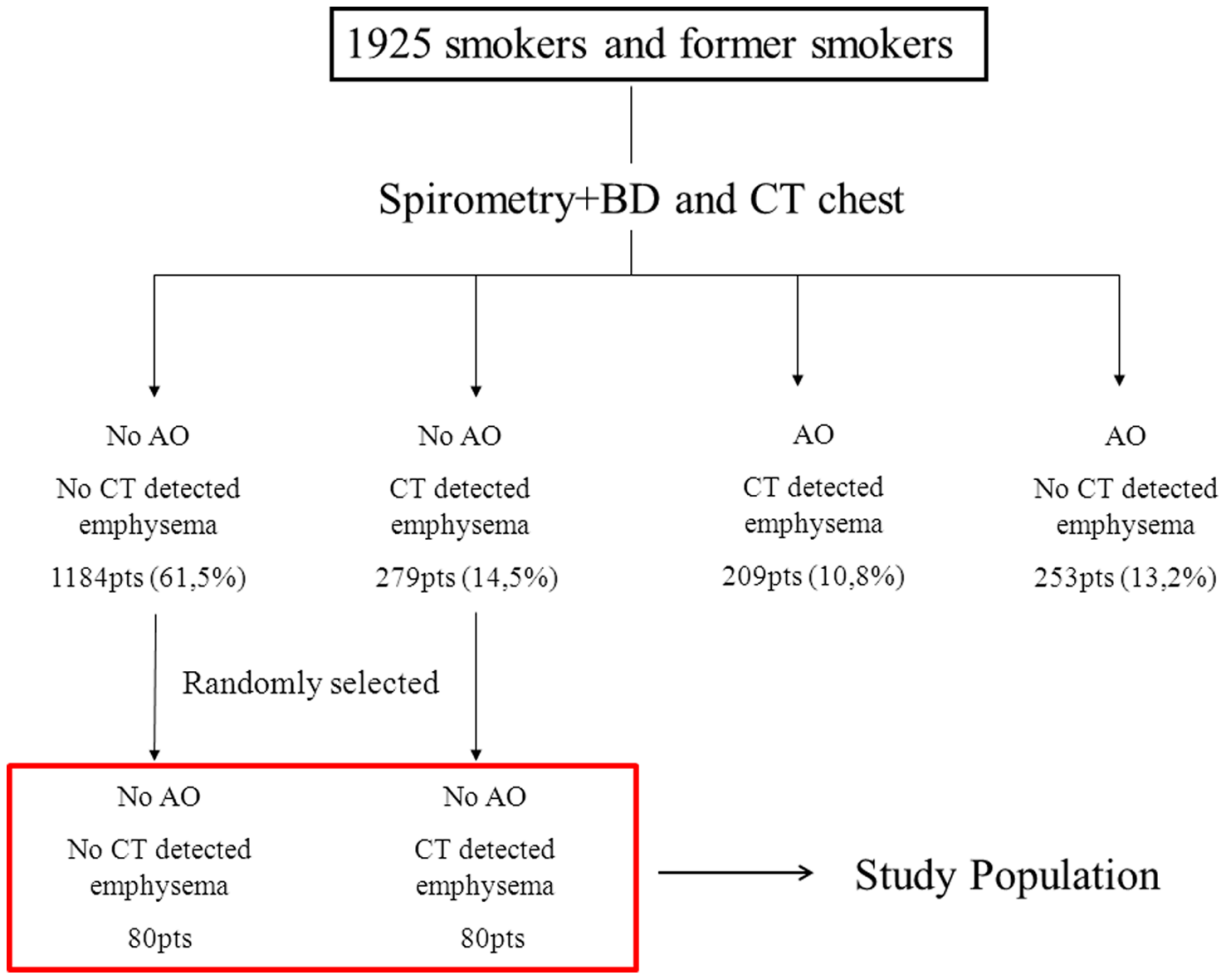
Flow chart showing the selection process of study participants.

Participants in this cross sectional observational study were randomly selected from a prospective cohort of individuals enrolled from September 2000 to December 2009 in a lung cancer screening study using low dose computed tomography (LDCT) [8]. Participants were at least 40 years old, had smoked 10 pack-years or more and had no symptoms of lung cancer. Details of the study protocol have been previously reported [9]. Briefly, participating individuals were offered a baseline LDCT of the chest followed by annual repeat studies for up to 10 years. All patients completed spirometry at enrolment and a baseline, standardized questionnaire including socio-demographic information and smoking history. From an initial sample of 1925 participants enrolled in the screening trial, we focused on those without airway obstruction on the initial spirometry. These individuals were further classified into two groups according to the presence or absence of emphysema on the LDCT (see flow chart in Figure 1). Thus, the study group had smokers and former smokers with emphysema on the LDCT but no evidence of airway obstruction, whereas the comparison group was made out of current and former smokers without emphysema and without obstruction. For the purpose of this study 80 individuals were randomly selected (http://www.randomizer.org/) from each of these two groups. Figure 1 shows the study flow chart with the selection process. The study protocol (IRB n°092/2009) was approved by the institutional ethics committee of the University of Navarra. All subjects signed an informed consent prior to participation.

### Low-Dose Computed Tomography

LDCT examinations were performed in a single breath-hold at end-inspiration with a multidetector CT scanner (Somatom Volume Zoom or Somatom Sensation 64, Siemens Medical Solutions, Forchheim, Germany) at low-radiation-dose settings (120 kVp, 20–40 mAs). All CT scans were reconstructed with 1.25-mm slice thickness and 1 mm intervals using a high spatial frequency reconstruction algorithm. Images were displayed at window settings appropriate for viewing the lung parenchyma (window width of 1,500 HU and window center of −650 HU). All images were read by two expert chest radiologists for visual assessment of the presence of emphysema, using validated criteria [10]. The extent of emphysema was graded ranging from no emphysema (0) to a maximum score of 4 indicating the presence of emphysema in more than 75% of the lung. For the purpose of this study, patients with a score ≥1 were classified as having emphysema.

### Spirometry Testing

Airway function was measured at baseline in all study participants using a flow spirometer (Vmax 22; SensorMedics, Yorba Linda, CA) according to American Thoracic Society standards [11]. Forced expiratory volume in the first second (FEV_1_) and forced vital capacity (FVC) values were expressed as a percentage of the predicted reference value according to the European Community Lung Health Survey [12]. Subjects with a postbronchodilator FEV_1_/FVC ratio <70% were classified as having airway obstruction following the criteria established by the Global Initiative for Chronic Obstructive Lung Disease (GOLD) [13].

### Cytokine Determinations

Plasma from all study participants was collected into Vacutainer® tubes. Blood specimens were centrifuged (2000×g), and aliquots stored at –80°C until laboratory analysis was performed. Six plasma samples (1 from those without emphysema) were not available or in appropriate conditions to be analyzed. Plasma levels of epidermal growth factor (EGF), interleukin 15 (IL-15), interleukin 1 receptor antagonist (IL-1ra), interleukin 8 (IL-8), macrophage chemotactic protein 1 (MCP-1), macrophage inflammatory protein 1 β (MIP-1β), tumor growth factor α (TGFα), tumor necrosis factor α (TNFα) and vascular endothelial growth factor (VEGF) were measured using MILLIPLEX™ MAP kit (Millipore, Billerica, MA, USA) with LUMINEX^®^ Xmap technology according to the manufacturer’s instructions [14]. The detection limits were 3.2 pg/mL, 2.5 pg/mL, 3.2 pg/mL, 2.04 pg/mL, 3.2 pg/mL, 3.2 pg/mL, 1.64 pg/mL, 2 pg/mL and 16.0 pg/mL, respectively. A LUMINEX^®^ 100 IS System (Luminex, Austin, TX, USA) was used to read the samples, compute standard curves and estimate cytokine concentrations. The intra-assay coefficient of variation for all assays was less than 10%. All the assays were carried out including samples for the four groups in analysis in each plate, and were randomly distributed, in order to minimise the effect of inter-assay variability.

Several studies have described markers such as TNFα, TGFα, IL-1ra and IL-8 as representative of the systemic inflammation present in patients with COPD/emphysema [15]. Other chemokines (IL-15, MCP-1, MIP-1β) and growth factors (EGF and VEGF) could be of interest for their putative role in the attraction and activation of leukocytes into inflamed tissue and could represent a systemic inflammatory link between emphysema and associated comorbidities such as lung cancer or cardiovascular disease. Therefore, biomarkers were selected based on these biological criteria and on the results from a study using high throughput proteomic analysis, in which patients with COPD/emphysema differed from smokers and non-smokers without airflow obstruction in the plasma levels of 25 biomarkers [16] associated with clinically relevant parameters.

### Statistical Analysis

Quantitative measurements are presented as mean (SD) for variables with normal distribution and median (IQR) for those not normally distributed. Categorical variables are shown along with their percentages. Comparisons between groups were made using Kruskal-Wallis test and Chi Square test for continuous and categorical demographic variables, respectively. Cytokine levels were not normally distributed and therefore were log-transformed for the purpose of comparison between groups and regression analyses. Univariate linear regression analyses were performed taking the log-transformed values of each plasma cytokine level as the dependent variable and individual variables (sex, age, pack-years and smoking status) as independent variables. Univariate analysis determines the strength of the association of emphysema with each of the cytokine level adjusting for factors that were statistical significant in the previous analysis: sex (EGF, MIP-1beta), age (VGF) and smoking status (TGFα). Significance was established as a two-tailed p-value ≤ 0.05 for all the analyses. Calculations were made using Stata statistical sotfware (version 10.1; Stata, College Station, TX).

## Results

One hundred and sixty current and former smokers participated in the study. Patient characteristics and physiologic measurements of both groups are shown in Table 1. Subjects were predominantly male. No differences were observed for age, cumulative tobacco exposure or smoking status at the time of the evaluation. Subjects with emphysema had lower absolute values of FEV_1_ but their predictive values of lung function were similar.

**Table 1:** Clinical and physiological characteristics of smokers and former smokers without airway obstruction: without emphysema vs. with emphysema.

	WithoutEmphysema(n = 0)	WithEmphysema(n = 80)	p
**Sex(males)**	56/79(70.9%)	61/80(76.2%)	0.73
**Age(SD)**	53.9(4.2)	52.3(8.4)	0.09
**Pack-years(SD)**	31.4(9.91)	34.2(9.9)	0.21
**Smoking(current)**	48/74(64.9%)	58/80(72%)	0.55
**FVC(L)(SD)**	3.87(0.75)	4.26(0.95)	0.03
**%FVC(SD)**	104.9(14.5)	106.4(13.8)	0.50
**FEV_1_(L)(SD)**	3.01(0.58)	3.23(0.74)	0.04
**FEV_1_%(SD)**	99(13.7)	99(12.8)	0.78

Table 2 shows groups comparison of their plasmatic cytokines levels. Compared to control smokers, individuals with emphysema had significantly lower log-transformed concentrations of IL-1ra, IL-8 and IL-15. Table 3 shows the univariate analysis for the different plasma cytokine concentrations and potential confounding variables (age, sex, pack years, and smoking status). Significant associations among them were found: EGF and MIP-1β with gender, VEGF with age, and TGFα with smoking status. Cumulative tobacco exposure was not associated with cytokine plasma levels.

**Table 2:** Plasma cytokines levels of smokers and former smokers without airway obstruction: without emphysema vs. with emphysema.

Cytokine	WithoutEmphysema(n = 80)	WithEmphysema(n = 80)	p
**EGF pg/mL**	157.5(100.2)	116.96(142.6)	0.06
**IL-15 pg/mL**	6.44(4.2)	4.88(3.63)	0.02
**IL-1rapg/mL**	19.23(12.62)	14.84(15.64)	0.04
**IL-8 pg/mL**	7.33(5.07)	6.63(4.38)	0.01
**MCP-1 pg/mL**	216.2(78.8)	210.43(91.67)	0.46
**MIP-1β pg/mL**	69.55(35.91)	68.73(41.39)	0.09
**TGF-α pg/mL**	3.18(4.62)	3.97(7.88)	0.27
**TNF-α pg/mL**	8.06(4.49)	6.95(4.23)	0.16
**VEGF pg/mL**	158.8(163.6)	163.0(216.9)	0.18

Cytokines levels are shown with their median and IQR (interquartile range).

**Table 3:** Univariate analysis for the different measured plasma cytokine levels.

Cytokine	Sex		Age		Pack-years		Smoking(current)	
	b	pvalue	b	pvalue	b	pvalue	b	pvalue
**EGF**	0.282*	0.01	−1.330	0.18	0.241	0.81	0.361	0.71
**IL-15**	−0.473	0.63	−0.194	0.84	−0.871	0.38	−1.172	0.24
**IL-1ra**	−0.064	0.12	0.201	0.84	0.332	0.74	−0.624	0.53
**IL-8**	0.445	0.65	−0.581	0.56	−0.021	0.98	−0.092	0.92
**MCP-1**	−0.710	0.47	−1.510	0.13	1.592	0.11	1.321	0.18
**MIP-1beta**	2.26*	0.02	0.071	0.94	0.092	0.92	−0.652	0.51
**TGFα**	0.210	0.83	−1.110	0.27	0.242	0.80	2.29*	0.02
**TNF-alfa**	1.542	0.12	0.362	0.72	0.782	0.43	−1.042	0.30
**VEGF**	0.111	0.98	−2.06*	0.04	1.502	0.13	1.652	0.10

* Statistically significant; b =  regression coefficient.

Cytokines levels were log transformed for the analysis.

Table 4 shows the multivariate analysis of each of the measured cytokines in the study group, using smokers without emphysema as a putative control group. Regression coefficients showed that, compared to smokers without emphysema, subjects with emphysema had lower levels of EGF (−0.317, p = 0.01), IL-15 (−0.219, p = 0.01), IL-8 (−0.180, p = 0.02), and IL-1ra (−0.220, p = 0.03). Interestingly, gender also had an influence on EGF (0.31, p = 0.01), IL-1ra (−0.19, p = 0.05) and MIP-1β (0.12, p = 0.03) levels, while age and smoking status had an influence on VEGF (−2.06, p = 0.04) and TGFα (2.29, p = 0.02), respectively.

**Table 4:** Multivariate analysis of each of the measured cytokines in the study group, using smokers without emphysema as a putative control group.

Cytokine	EmphysemaNoAirwayObstruction	
	b	p value
**EGF**	−0.317*	0.01
**IL-15**	−0.219*	0.01
**IL-1ra**	−0.220*	0.03
**IL-8**	−0.188*	0.02
**MCP-1**	−0.012	0.83
**MIP-1beta**	−0.068	0.26
**TGF-alfa**	0.234	0.10
**TNF-alfa**	−0.137	0.08
**VEGF**	0.039	0.75

* Statistically significant; b =  regression coefficient.

Analysis adjusted for sex (EGF, MIP-1beta), age (VEGF) and smoking status (TGF-alfa) because these factors were statistically significant in the univariate analysis.

Cytokines levels were log transformed for the analysis.

## Discussion

The main finding of this study is that in current and former smokers without airway obstruction on a spirometry, the presence of emphysema detected on a CT is associated with changes in levels of certain cytokines. The differences found include lower levels of certain cytokines that may result in activation of apoptosis (EGF, IL-15) and altered immunosurveillance (IL-15, IL-1ra).

The presence of an abnormal systemic inflammatory response in some patients with stable COPD is a well-recognised phenomenon [17], [18], [19]. However, little is known about the specific impact airway obstruction or emphysema may have on this systemic inflammatory response. Bon et al. [19], published data regarding peripheral blood biomarker patterns in subjects with CT detected airway thickening and emphysema. The study enrolled two hundred and thirty four current and former smokers participating in a Lung Cancer Screening Study. Each subject underwent spirometry and chest CT imaging. Thirty-three different chemokines and growth factors were measured in serum. The authors found that certain inflammatory biomarkers were associated with distinct phenotypic expressions of disease such as airway remodelling or CT detected emphysema. In particular, emphysema defined by parenchymal voxels with computed attenuation values less than −950 Hounsfield Units (HU) was associated with higher blood levels of IL-6 and MMP-7, but lower TNFα levels. In the present work we did not find any association between emphysema and TNFα levels, and IL-6 and MMP-7 were not determined. Papaioannou et al. [20], also reported that COPD patients with CT detected emphysema show an increased systemic oxidative stress response and harbour elevated fibrinogen levels, suggesting that clinical differences in the phenotypic expressions of tobacco related pulmonary disease correlate with discordant inflammatory response profiles. The methodology of our study is somewhat different since we chose to focus on the effect of emphysema, and thus selected only individuals without airway obstruction for both the study and the control groups [19], [20]. Several authors including our group have reported that a significant proportion of individuals participating in lung cancer screening studies have emphysema without airway obstruction [5], [6], [21]. We believe that this particular group of individuals provide an opportunity to study potential mechanisms uniquely involved in the development of emphysema.

EGF is a soluble growth factor implicated in the regulation of airway mucus secretion, repair, angiogenesis, and remodelling [17]. Recent data from research performed in the homozygous mutant klotho (KL[−/−]) mouse suggest that increased apoptosis of airway cells via the inhibition of the EGF-dependent pathway may be involved in the development of the aging lung process that includes emphysema [22]. Moreover, EGF receptor serum levels are reduced in individuals with non-small cell lung carcinoma [23], while mutations of the EGF receptor have led to targeted therapies in the treatment of lung adenocarcinoma [24]. Our findings of decreased levels of EGF in patients with emphysema are in line with this interesting bio-pathological pathway [25].

IL-15 is a pluripotent antiapoptotic cytokine implicated in both the innate and adaptive immune response to viral infection and cancer, and has been postulated as a highly promising immunomodulatory agent in cancer therapy [25]. IL-15 regulates cell proliferation, survival, and function of NK cells. Plasma levels of this cytokine are reduced in smokers [26]. IL-15 also favours muscle fibre hypertrophy and antagonises the muscle protein wasting typically seen in patients with cancer [27] or with emphysema [28].

The lower levels of IL-15 found in subjects with emphysema could possibly reflect impaired tumour immunosurveillance and thus be implicated in their well-known increased risk of lung cancer [29].

IL-1ra is an anti-inflammatory protein synthesised by hepatocytes that downregulates the inflammatory response [30]. Studies in transgenic and knockout mice indicate that IL-1ra is an important component of the host defence against endotoxin-induced injury and plays a key role in the modulation of IL-1 activity, a potent stimulator of metalloproteinases [31]. IL-1 suppression by IL-1ra results in a long-term beneficial effect in chronic inflammatory diseases [31]. Depressed levels of this cytokine in patients with emphysema might imply an inability to downregulate the chronic inflammatory response. Other investigators have reported similar findings in smokers [32] and in patients with COPD [33]. Interestingly, IL-1ra gene polymorphisms have been linked in a Norwegian study to an increased risk of non-small lung cancer [34].

IL-8 is a neutrophil chemoattractant and activator that induces a rise in intracellular calcium concentration and exocytosis with release of enzymes and proteins from intracellular organelles. It also has chemoattractant properties for T-cells. Up to 43% of the chemotactic activity of sputum can be attributed to IL-8 [15]. In patients with COPD, IL-8 levels are usually increased. In our study, levels of IL-8 were lower in individuals with emphysema, suggesting that the mechanisms involved in the pathogenic process are different than in patients with airflow obstruction.

### Study Limitations

Our study is limited by the relatively small number of subjects enrolled; although to our knowledge it is the only study thus far that evaluates the inflammatory profile of patients with emphysema but without COPD according to current accepted criteria. We believe it to be hypothesis-generating work, which needs to be reproduced in larger populations and perhaps with more mechanistic methodologies. Errors in data interpretation are another important limitation of any cross-sectional study. Specific cytokine profiles, for example, may be viewed as either a cause or a consequence of a given phenotypic expression of a disease. It is impossible to know which is which without additional longitudinal studies investigating cytokine profiles in subjects encompassing the entire spectrum of disease severity.

### Conclusions

In conclusion, our study suggests that current and former smokers with CT-detected emphysema and normal spirometry have a distinct cytokine profile when compared to subjects without emphysema. Lower levels of EGF, IL-15, and IL-1ra may be involved in the activation of apoptosis or in the impairment of immunosurveillance, two mechanisms that lead to a higher lung cancer risk. Larger prospective studies are needed to support our findings, and to investigate whether discordant cytokine profiles might account for variations in the systemic manifestations within a given population.
